# Wearable Technology for Chronic Wound Monitoring: Current Dressings, Advancements, and Future Prospects

**DOI:** 10.3389/fbioe.2018.00047

**Published:** 2018-04-26

**Authors:** Matthew S. Brown, Brandon Ashley, Ahyeon Koh

**Affiliations:** Department of Biomedical Engineering, State University of New York at Binghamton University, Binghamton, NY, United States

**Keywords:** wound healing, chronic wound, wound monitoring microsystem, flexible and stretchable biosensors, biomarkers for wound care

## Abstract

Chronic non-healing wounds challenge tissue regeneration and impair infection regulation for patients afflicted with this condition. Next generation wound care technology capable of *in situ* physiological surveillance which can diagnose wound parameters, treat various chronic wound symptoms, and reduce infection at the wound noninvasively with the use of a closed loop therapeutic system would provide patients with an improved standard of care and an accelerated wound repair mechanism. The indicating biomarkers specific to chronic wounds include blood pressure, temperature, oxygen, pH, lactate, glucose, interleukin-6 (IL-6), and infection status. A wound monitoring device would help decrease prolonged hospitalization, multiple doctors' visits, and the expensive lab testing associated with the diagnosis and treatment of chronic wounds. A device capable of monitoring the wound status and stimulating the healing process is highly desirable. In this review, we discuss the impaired physiological states of chronic wounds and explain the current treatment methods. Specifically, we focus on improvements in materials, platforms, fabrication methods for wearable devices, and quantitative analysis of various biomarkers vital to wound healing progress.

## Introduction

Chronic wounds, those that exceed a 3-month healing process or that fail to demonstrate improvement through treatment under the normal physiological tissue repair mechanism in a timely manner, present severe difficulties for individuals afflicted with this condition and a substantial financial burden for the healthcare industry (Ochoa et al., 2014). In the US alone, chronic wounds annually cost $20 billion and affect 5.7 million people (Järbrink et al., 2017). These numbers will continue to increase with the aging population more likely to develop diabetes mellitus (Sen et al., 2009). While any skin lesion can potentially become a chronic wound, impaired physiological states and various diseases can alter the wound healing process. Debilitations in tissue repair can be due to vascular insufficiency, diabetes mellitus, aging, nutritional defects, mechanical stress, prolonged internal pressure, and autoimmune diseases, as well as biochemical abnormalities and underlying physiological issues (Eming et al., 2014; Frykberg and Banks, 2015). Furthermore, the prolonged open wound poses an infection risk, as it can easily be populated by various bacteria from the external environment, eventually leading to biofilm formation. As such, chronic wounds lead to adscititious problems and complications.

Current common treatment of chronic wound relies on wound dressings tailored to the state of the wound; often described as deep or shallow, clean or infected, and dry or exudative (Abrigo et al., 2014). Various wound dressings have been engineered to fit specific wounds based on the wound's characteristics. Of note, chronic wounds exhibit many symptoms which limit wound dressing treatment outcomes because they have been developed for treating a finite number of wound conditions concurrently. Other therapeutic regimens are available including surgical approach and negative pressure therapy. Although the advances in biomaterials and biomedical devices are making great strides, there is still a lack of understanding in the underlying molecular basis involved in failed tissue repair. Moreover, a lack of analytical assessments for wound diagnosis significantly impedes clinical success. Therefore, a biomedical device capable of monitoring wound parameters noninvasively at the wound site, with the use of a closed loop therapeutic system offers an alternative treatment approach for effective wound management to decrease healing time and mortality rates (Mehmood et al., 2014).

In this review, the impaired physiological response of chronic wounds is explained, and the current wound care technologies are described. Specifically, we focus on the next generation of wound monitoring microsystems. The advanced materials and fabrication methods for wearable devices, and the quantitative analysis of various biomarkers that are important to evaluate wound healing process are presented. In addition, we discuss the current challenges the field faces as well as the future work needed to be done.

## Wound repair physiology

**Figure 1:**
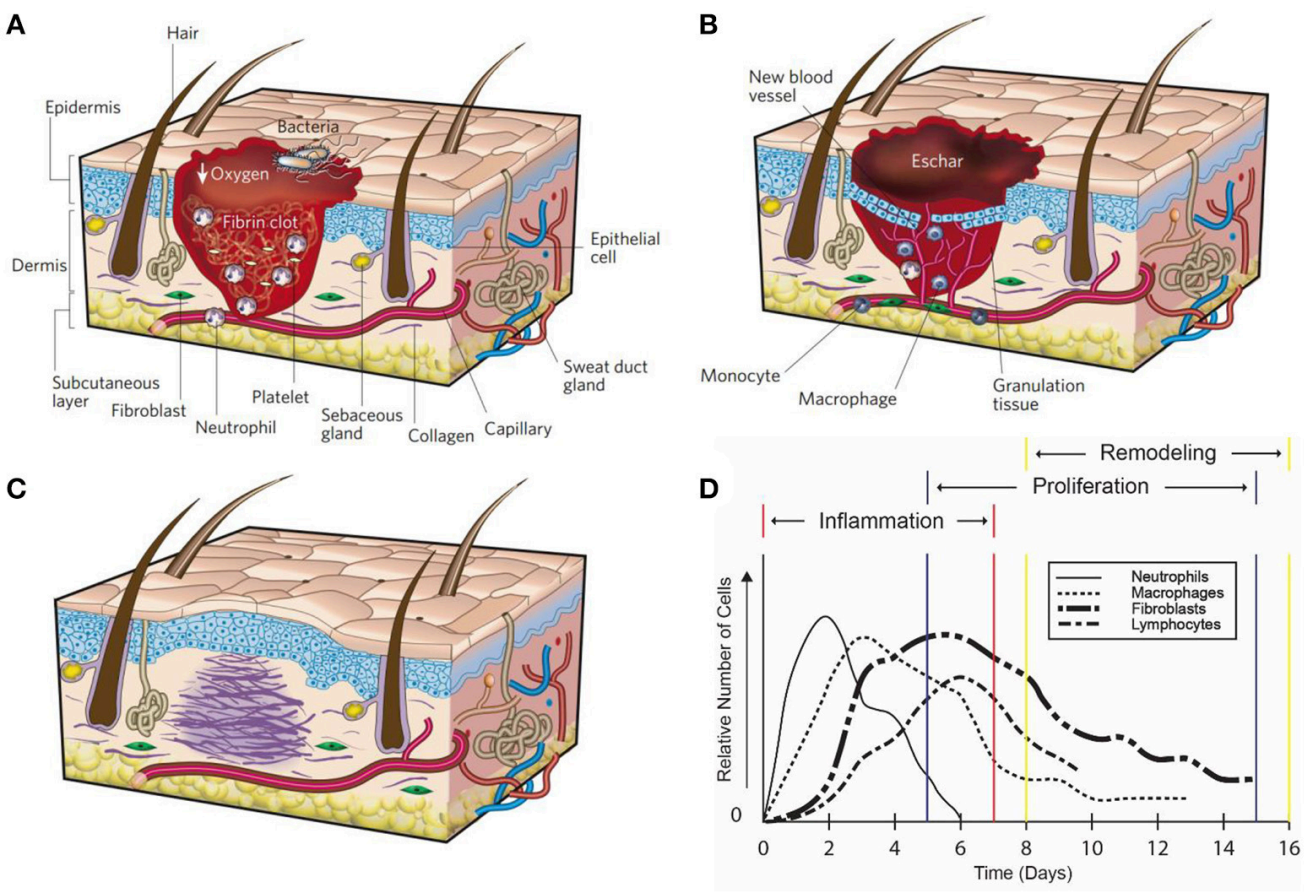
Wound healing. The three wound repair stages: **(A)** inflammation, **(B)** proliferation, and **(C)** remodeling. Reprinted with permission from Gurtner et al. (2008). **(D)** The time course of cell migration for immune cells during wound healing (Witte and Barbul, 1997).

In response to injury multicellular organisms exhibit tissue repair through a complex cascade of molecular mechanisms resulting in a fully healed wound. The wound healing process can be classified into three overlapping but distinct stages: (1) hemostasis and inflammation, (2) proliferation, and (3) remodeling (Figures 1A–C) (Witte and Barbul, 1997; Gurtner et al., 2008; Eming et al., 2014).

### Hemostasis and inflammation

Immediately after tissue damage occurs, hemostasis initiates. The blood vessels exposed to the injury site constrict, platelets aggregate at the site and form a platelet clot, followed by a fibrin matrix which is cleaved from fibrinogen during the coagulation of blood (Gonzalez et al., 2016). The fibrin matrix is more durable to the outside environment, forms a barrier to prevent microorganisms from entering, and acts as a scaffold to help organize cells throughout the healing process (Gonzalez et al., 2016). The initial hemostasis process takes about 3–5 min to form depending on the concentration of platelets in the blood. The formation of the platelet plug and fibrin matrix leads to the release of cytokines and growth factors which elicit an inflammatory reaction that is necessary to tackle foreign bodies and progress tissue repair. Neutrophils initiate the inflammatory response while first arriving at the wound site due to chemokines released during hemostasis (Figure 1D) (Jhunjhunwala et al., 2015). After 2–3 days, monocytes migrate into the wound in response to chemokines and differentiate into macrophages. The main functions of macrophages are phagocytosis, angiogenesis, cell recruitment and activation, regulate wound debris (i.e., phagocytosis and enzymes), and wound matrix synthesis. Macrophages synthesize nitric oxide early in the healing process, which has antimicrobial properties, induces angiogenesis, increase collagen deposition, and improves scar mechanical strength (Witte and Barbul, 1997; Park and Barbul, 2004; Luo and Chen, 2005). The inhibition of nitric oxide has been shown to impair reepithelialization and reduce collagen deposition (Witte and Barbul, 1997; Park and Barbul, 2004). Proinflammatory cytokines secreted by neutrophils and macrophages, such as interleukin-6 (IL-6), interleukin-1 (IL-1β), and tumor necrosis factor alpha (TNF-α), help to delay the wound healing process (Gabay, 2006; Eming et al., 2014). Therefore, a deficiency of neutrophils and macrophage cells is responsible for the inhibition of the inflammatory response phase (Gurtner et al., 2008).

During chronic wound healing, inflammation is prolonged and alters the progression of wound healing. Proinflammatory cells result in the lingering inflammatory phase. The multiprotein complex inherent to the immune system continuously releases IL-6 and IL-1β from neutrophils and macrophages during the formation of chronic wounds. IL-6 plays a detrimental role in tissue repair by eliciting the release of monocyte chemoattractant protein-1 (MCP-1) and monocyte chemoattractant protein-2 (MCP-2), which recruits monocytes to maintain the inflammatory response and modify the immune system respectively. Therefore, elevated IL-6 levels in wound tissue can correlate to markers of the inflammatory phase (Gabay, 2006). Additionally, elevated metalloproteinases due to proinflammatory cytokines (IL-1β and TNF-α) have been shown to degrade the extracellular matrix (ECM), diminishing cell migration at the wound site (Eming et al., 2014). Consequently, neutrophils and macrophages remain at the wound site, releasing harmful proinflammatory cytokines, and increasing metalloproteinase enzyme rates in response to elevated IL-6 levels. Furthermore, the rate in which metalloproteinase enzymes degrade proteins is directly related to the wound pH and temperature. Chronic wounds exhibit a pH around 7.15–8.90 at the wound bed, creating a slightly basic environment (Gethin, 2007). Metalloproteinase enzymes degrade proteins more rapidly in basic conditions, consuming more oxygen from the tissue to speed up the process. Therefore, it is clinically favorable to have a more acidic environment to slow metalloproteinase enzyme degradation rates, decrease abnormal collagen in the wound bed, increase fibroblast activity, and enhance the toxicity of the environment to bacteria for effective wound treatments (Gethin, 2007). A high wound temperature increases the enzymatic, chemical reaction rate and can be used as an indicator of a prolonged inflammatory phase. In general, a prolonged temperature increase greater than 1.11°C (Fierheller and Sibbald, 2010) at the exposed wound site can indicate infection due to the presence of bacterial organisms (Salvo et al., 2017). Chronic wounds often suffer from tissue hypoxia, disrupting the normal physiological healing mechanism on the molecular level, further inhibiting the finalization of the inflammatory phase due to the lack of oxygen present at the wound. During sustained inflammation, the partial pressure of oxygen (pO_2_) remains around 5–20 mmHg in the exudate of chronic wounds, whereas healthy tissue exhibits 30–50 mmHg (Schreml et al., 2010). The tissue hypoxia results in an alternation of the metabolic activity as mitochondria produce less adenosine triphosphate (ATP), causing the release of additional proinflammatory cytokines (e.g., IL-6, IL-1β, and TNF-α) (Schreml et al., 2010). Few studies have examined wound fluid from the wound site, reporting exudate contains biochemical information composed of growth factors and chemokines, which help to transition from the inflammatory phase to the proliferative phase and aid in collagen production, angiogenesis, and overall wound closure (Banda et al., 1982; Pricolo et al., 1990; Regan et al., 1991). Chronic wounds often remain in a prolonged inflammatory phase and lack the ability to transition into the subsequent phases to complete the wound healing process, resulting in an open wound.

### Proliferation

The second stage, proliferation, occurs 2–10 days after injury (Gurtner et al., 2008). Following the inflammatory phase, macrophages and neutrophils release various cytokines and chemokines to recruit the necessary cells for proliferation at the wound bed. Keratinocytes, fibroblast, lymphocytes, myofibroblast and endothelial cells are involved in the proliferation phase. Keratinocytes aggregate at the wound edge and cover the wound bed to restore the skins barrier function (Das and Baker, 2016). Fibroblast become activated in the surrounding tissue by platelet-derived growth factor (PDGF) and epidermal growth factor (EGF), which induce chemotaxis and proliferation of the fibroblast. After fibroblasts migrate to the wound site from surrounding tissue, they begin to secrete collagen, fibrinogen, and ECM to replace the fibrin matrix (Das and Baker, 2016). This forms granulation tissue, which is imperative for proper tissue repair and angiogenesis. New blood vessels then form through angiogenesis and create new capillaries from existing blood vessels (Eming et al., 2014; Das and Baker, 2016). Blood vessel formation is essential as it supplies the wound site with water, nutrients, and oxygen that is crucial for cell proliferation. Epithelial cells proliferate in excisional wound healing in order to reestablish a barrier to the external environment to prevent fluid loss and infection (Witte and Barbul, 1997). A scar is constructed as the wound closes from the edges while the fibroblast secretes collagen and extracellular matrix.

If chronic wounds surpass the inflammatory phase, during the proliferative phase, the hypoxia at the wound further impairs the healing mechanism associated with the collagen deposition. Fibroblasts require a partial pressure of oxygen about 30–40 mmHg to maintain the natural collagen synthesis production (Schreml et al., 2010). Additionally, fibroblasts become impaired when lactate concentration in the wound increases above 7 mM, whereas normal tissue lactate levels are around 1–3 mM (Löffler et al., 2011). Furthermore, the increase in lactate levels assists in making the wound more susceptible to bacterial contamination, which can revert the healing process back to the inflammatory phase (Löffler et al., 2011).

### Remodeling

The final stage, remodeling, occurs 2–3 weeks after injury and can last for at least a year (Gurtner et al., 2008). During this stage, the processes initiated by the immune response slows down and is then terminated. The endothelial cells, macrophages, and neutrophils undergo apoptosis leaving behind a collagen and protein extracellular matrix in the wound bed. Epithelial interactions continue to reestablish the normal mechanical properties of skin and maintain homeostasis. The deposition of the collagen in the wound occurs during reepithelization. This phase is important as the quality, rate, and total amount of deposition of collagen determines the strength of the scar. An impaired inflammatory phase, such as in diabetes, results in poor matrix deposition and a scar with reduced mechanical strength (Witte and Barbul, 1997).

Chronic wounds are considered as an ideal environment for biofilms as the necrotic tissue allows for suitable bacterial attachment. Of note, 60 percent of all chronic wounds develop a biofilm through the cyclic spreading and shedding of planktonic bacteria (Zhao et al., 2013). Bacteria attach to the wound site, multiply, develop microcolonies, and produces a substance similar to ECM called extracellular polymeric substances (EPS) to create a biofilm. Once developed, it leads to persistent infection and becomes difficult to eradicate. Indeed, biofilm bacteria show greater tolerance to antibiotic treatment. Chronic wounds are typically populated with *Pseudomonas aeruginosa* (*P. aeruginosa*) and *Staphylococcus aureus* (*S. aureus*) developing into a biofilm and infecting the wound tissue (Hill et al., 2010). *P. aeruginosa* wounds have been shown to increase the presence of neutrophils, whereas *S. aureus* augments the proinflammatory cytokines. Additionally, macrophages become overwhelmed with the vast bacterial population persistence resulting in increased inflammation, macrophage presence, wound pH, and temperature at the wound site. The lingering presence of a biofilm also consumes vital oxygen and nutrients from the wound, depriving essential cells, impairing the immune response in combating the infection and regenerating the damaged tissue, further elongating the tissue healing time.

## Wound dressings

Currently, the standard of care for chronic wounds is through the application of wound dressings, which call for regular replacement as well as constant monitoring from a healthcare professional. Wound dressings are utilized to accelerate tissue regeneration at the wound, prevent and treat infections, and restore the skins native physiological properties. The major advancement made of modern wound dressings relies on the capability of maintaining moisture in the wound environment. Dressings that help regulate moisture have been shown to increase tissue regeneration rates, decrease pain and infection, and improve the appearance of the wound (Fonder et al., 2008).

In addition to sustaining moisture, other biomedical engineering strategies have sought to provide an optimal healing environment. The requirements include being non-toxic, protecting the wound from the external environment such as debridement and mechanical stress, exhibiting free fluid and gas exchange, allowing absorption of wound exudate, being easily removable without disturbing underlying tissue, protecting against infection and bacterial infiltration, demonstrating thermal insulation, and requiring minimal replacement frequency of the dressing (Abrigo et al., 2014; Andreu et al., 2015). The wound dressings can be classified into four categories: passive, interactive, advance, and bioactive. Passive dressings include the simplest of dressings, such as cloth gauze, providing a slight barrier against mechanical stress. However, this type of material can adhere to the wound bed causing frequent and difficult replacement (Abrigo et al., 2014). Unlike passive dressings, interactive dressings contain polymeric films and/or foams, making them gas and fluid permeable while providing a barrier against bacterial invasion (Zahedi et al., 2010). Advanced dressings maintain wound moisture, which improves the kinetics of tissue regeneration. Materials such as hydrocolloids and alginates are examples of the advanced dressings capable of absorbing moderate wound exudate. The fourth type of dressing, bioactive, are those which include biological scaffolds or drug delivery systems, and play an active role in the regeneration of wound tissue. Bioactive dressings draw significant attention in current wound care research initiatives; capable of being the next important development in healing chronic wounds (Abrigo et al., 2014). Indeed, with personalized medicine on the rise, patient-specific wound dressing systems that depend on the characteristics associated with each wound have the potential for future medical care.

The modern approach for identifying wound dressings depend on the physiological state of the wound. Important characteristics such as healing stage, moisture levels, exudate volume, and current infection status are necessary to consider in the application of providing the optimal dressing in accordance with each wound. Various wound dressing materials have been engineered for providing an effective treatment as well as holding great potential to be developed as the substrate for wearable wound monitoring devices (Table 1).

**Table 1:** Various wound dressings.

**Category**	**Dressings**	**Appropriate for:**	**Not suitable for:**	**Porous**	**Moisture retention**	**Market availability**
Passive	Gauze^a^	Dry shallow wounds	Wound protection	No	No	Yes
Interactive	Foams^b^	Heavy wound exudate, deep wounds	Dry wounds	Yes	Yes	Yes
	Transparent films^c^	Moisture retention, wound protection	Wound exudate absorption	No	Yes	Yes
	Hydrogels^d^	Wound protection, minor wound exudate, long wear time	Moderate wound exudate	Yes	Yes	Yes
Advanced	Alginates^e^	Heavy wound exudate	Necrotic tissue	Yes	No	Yes
	Hydrocolloids^f^	Minor wound exudate, Moisture retention	Dry wounds, heavy wound exudate, necrotic tissue, infected wounds	Yes	Yes	Yes
Bioactive	Tissue engineered skin^g^	Large wounds, severe burns	Infected wounds, necrotic tissue	Yes	No	Yes

a *Fonder et al. (2008) and Abrigo et al. (2014)*.

b *Fonder et al. (2008) and Ochoa et al. (2014)*.

c *Fonder et al. (2008) and Abrigo et al. (2014)*.

d *Fonder et al. (2008) and Abrigo et al. (2014)*.

e *Fonder et al. (2008) and Abrigo et al. (2014)*.

f *Fonder et al. (2008); Abrigo et al. (2014), and Ochoa et al. (2014)*.

g *Abrigo et al. (2014) and Han and Ceilley (2017)*.

### Passive dressings

Gauze, the most common and least expensive dressing made of cotton, is used in treatment for flat, shallow wounds that do not secrete wound exudate. However, gauze dries out the wound and can bind to the wound over time, creating difficulties during the removal and replacement process. Furthermore, gauze only provides the wound with limited protection against bacterial invasion. Dry gauze may degrade reepithelization and the effectiveness of the inflammatory response by altering the oxygen environment under the dressing (Han and Ceilley, 2017). Additionally, gauze can also be impregnated with a variety of substances to enhance the healing capabilities of the dressing. The modern dressing form of gauze is pretreated with moisturizing agents to control hydration at the wound to provide faster healing times than plain dry gauze. Petrolatum implemented into gauze offers less drying at the wound site and yields easier replacement of the dressing due to less adherence to the wound (Fonder et al., 2008).

### Interactive dressings

Foam or sponge dressings provide an alternative to gauze, as they can absorb heavy wound exudate that is suitable for deep wounds, diabetic ulcers, minor burns, and venous insufficiency ulcers. Polyurethane absorptive foam dressings create a hydrophilic interface at the wound interaction site, and a hydrophobic surface faced to the outside environment to provide gas and fluid permeability while protecting against bacterial invasion. Additionally, foam dressings can be used in conjunction with other dressings, such as hydrogels to enhance the reepithelization at the wound. When applied with slight pressure, it has been shown to reduce over-granulation (Abdelrahman and Newton, 2011). Of note, the highly absorbent nature of the dressing maintains low adherence and a painless removal process (Abdelrahman and Newton, 2011). Consequently, they are not suitable for eschar-covered or dry wounds and arterial ulcers due to high absorptivity (Das and Baker, 2016).

The dressings consisting of transparent polyurethane films are thin and highly flexible, capable of maintaining a proper gaseous and fluid transport while remaining impermeable to water and bacteria. The transparency of the film allows for clinicians to visualize the wound healing process. Depending on the material composition, wound moisture and vapor transmission rate can be controlled, allowing for personalized alternatives depending on the wounds status (Jones et al., 2006). However, such dressings may not be suitable for wounds that exhibit heavy exudates due to fluid trapping and maceration (Fonder et al., 2008). Additionally, the adhesive nature of the dressing may cause complications during removal resulting in skin tears (Das and Baker, 2016).

Hydrogels maintain the moisture level in the wound environment, their underlying mechanism is constituted by a hydrophilic polymer containing a swelled water composition. Dry wounds that produce little to no exudate levels are best suited for such dressings as they pose the ability to donate moisture and rehydrate necrotic tissue. Additionally, they are highly effective at providing the wound with autolytic debridement in deep, necrotic wounds, while maintaining a moist environment (Jones and Vaughan, 2005). Hydrogels can be applied to the wound bed and are removed in a simple manner, with little to no pain. Furthermore, the cooling sensation provided with hydrogels assists in associated chronic wound pain relief for patients (Fonder et al., 2008).

### Advanced dressings

Alginate dressings are highly absorbent and suitable for wounds with heavy exudate levels. An exudative wound environment allows alginate dressings to function. The sodium secreted from the wound is exchanged for the calcium released from the alginate, forming a gel. The release of calcium ions helps to maintain wound homeostasis through platelet activation, preventing the wound from constant bleeding. Additionally, the hydrophilic character of alginate contributes to moisturizing the wound and facilitating reepithelization (Fonder et al., 2008; Skórkowska-Telichowska et al., 2013).

Hydrocolloid dressings are a hydrophilic gel which provides improved wound exudate absorption in contrast to hydrogel dressings. The main application for hydrocolloids is through minor exudate management, whereas large exudate levels can cause separation from the dressing and the wound bed. The anaerobic environment provided by such dressings has been shown to improve hypertrophic graduation of tissue. Additionally, hydrocolloid dressings are engineered with a variety of proteins, adhesives, and polysaccharides to enhance the tissue regeneration rate. But prolonged wearability has been shown to induce allergic contact dermatitis (Schultz et al., 2003).

### Bioactive dressings

Tissue engineered dressings can restore the native healing process using endogenously incorporated chemical signals (i.e., growth factors, differentiation factors, and cell adhesion molecules) (Singh et al., 2008) and providing a material matrix similar to ECM. The ideal dressing ought to be able to promote regeneration of tissue by attracting cells which can induce tissue and blood vessel synthesis, initiate cellular proliferation, and provide a matrix to organize cells and assist in new tissue deposition (Gould, 2016). Clinically, decellularized dermal matrices have been used as wound dressings for burn regeneration, foot wounds, and venous stasis ulcers. Additionally, dressings seeded with the patient's own stem cells through the culturing of epithelial autografts have had some success in resurfacing burns (Wong and Gurtner, 2012). The loss of epidermal stem cells at the wound edges has been proposed to be one of the physiological factors affecting tissue repair for chronic wounds (Wong and Gurtner, 2012). Clinically, several studies have proven the efficacy of tissue engineered dressings, despite being a costly alternative, they present accelerated healing mechanisms which reduce hospital stay and decreases hospital-associated infection risk (Han and Ceilley, 2017). Commercially, there is an abundance of tissue engineered dressings available for treatment options (e.g., Alloderm, Apligraf, Biobrane, Bioseed, Dermagraft, etc.) (Han and Ceilley, 2017).

### Antimicrobial dressings

Chronic wounds, often infiltrated and overwhelmed by bacterial colonization, require antimicrobial agents to prevent contamination, colonization, and infection from bacteria. Bacterial colonization of 10^2^–10^3^ colony-forming units (CFUs) per gram of tissue has been shown to induce trauma at the wound, whereas more than 10^6^ CFUs results in the inability to heal through normal physiological mechanisms (Schultz et al., 2003). Clinically, quantitative analysis of bacterial colonization and infection requires a tissue biopsy, which is costly and can often further damage the wound. Antimicrobial and antibiotic treatments are commonly used to eradicate bacteria at the wound site. However, application of such treatments on wounds with an absence of a bacterial biofilm presents a toxic wound environment, necrosis of tissue, and ultimately an impaired tissue repair process (Lo et al., 2009). Antibiotics can be topically applied to the wound in conjunction with dressings, but due to the rise of antibiotic-resistant bacteria, this will be an ineffective alternative. The two most widely used types of antimicrobial dressings utilize silver and iodine. Although actively used by clinicians, the effectiveness of silver-dressings has yet to provide significant evidence in reducing bacterial infection (Lo et al., 2009). Furthermore, *in vitro* studies have shown silver to be toxic to essential wound healing cells such as keratinocytes and fibroblast. The consensus of the effectiveness of silver-releasing dressings is still unclear as some studies have shown an acceleration in wound healing (Schultz et al., 2003; Fonder et al., 2008). In contrast, *in vitro* models have shown iodine dressings to be effective against biofilms (Hill et al., 2010). However, the effectiveness of silver and iodine incorporated dressings are still uncertain. Nonetheless, with the threat of antibiotic-resistant bacterial strains on the rise, preventing infection at the wound site is crucial.

### Current research

While various wound dressings are available, very few dressings have shown substantial evidence in stimulating significant tissue repair (Chaby et al., 2007). Current research has focused on advanced biological therapies incorporated into dressings. Growth factors are essential in the mediation of major cellular activities throughout the healing cascade, and thus growth factor therapies assume that a cellular deficiency causes impaired healing in chronic wounds (Schultz et al., 2003; Fonder et al., 2008). The current standard of care therapy for full-thickness cutaneous wounds is recombinant human platelet-derived growth factor-BB (rhPDGF-BB, Becaplermin), which is the only topical growth factor available to clinicians in the US (FDA approved), with its intended use for diabetic neuropathic ulcers (Frykberg and Banks, 2015). In numerous clinical trials, rhPDGF-BB has been shown to help in the treatment of chronic lower extremity diabetic ulcers by increasing the incidence of complete healing as well as functioning as a safe therapy that is well tolerated (Steed and Study Group, 1995; Smiell et al., 1999). Naltrexone (NTX) is an alternative therapy that is FDA approved for systemic application and is currently being evaluated for topical treatment (McLaughlin et al., 2017). Internationally, Trafermin (AdisInsight, 2016) and Nepidermin (AdisInsight, 2015) are also used for the treatment of decubitus wounds but are not FDA approved for clinical use in the US. Furthermore, active therapeutic methods have been evaluated such as negative pressure therapy (Fonder et al., 2008; Ubbink et al., 2008) and hyperbaric oxygen therapy (Fonder et al., 2008; Frykberg and Banks, 2015). Both have had varying success at proving accelerated tissue regeneration and more trials are needed to assess their efficacy.

Although both passive and active strategies for wound treatments and biocompatible materials have been studied, the current care system lacks a feedback mechanism that monitors the dynamics of the physiochemical microenvironment so treatment can be improved. A multifunctional *in situ* device, which can diagnose wound parameters, treat various chronic wound symptoms, and reduce infection at the wound; a point-of-care (POC) system on a wearable platform has the potential to improve current treatment options. In developing a wound monitoring microsystem, current wound dressing research and materials can indeed be incorporated into sensor substrates or platforms.

## Advanced wearable biosensors

**Figure 2:**
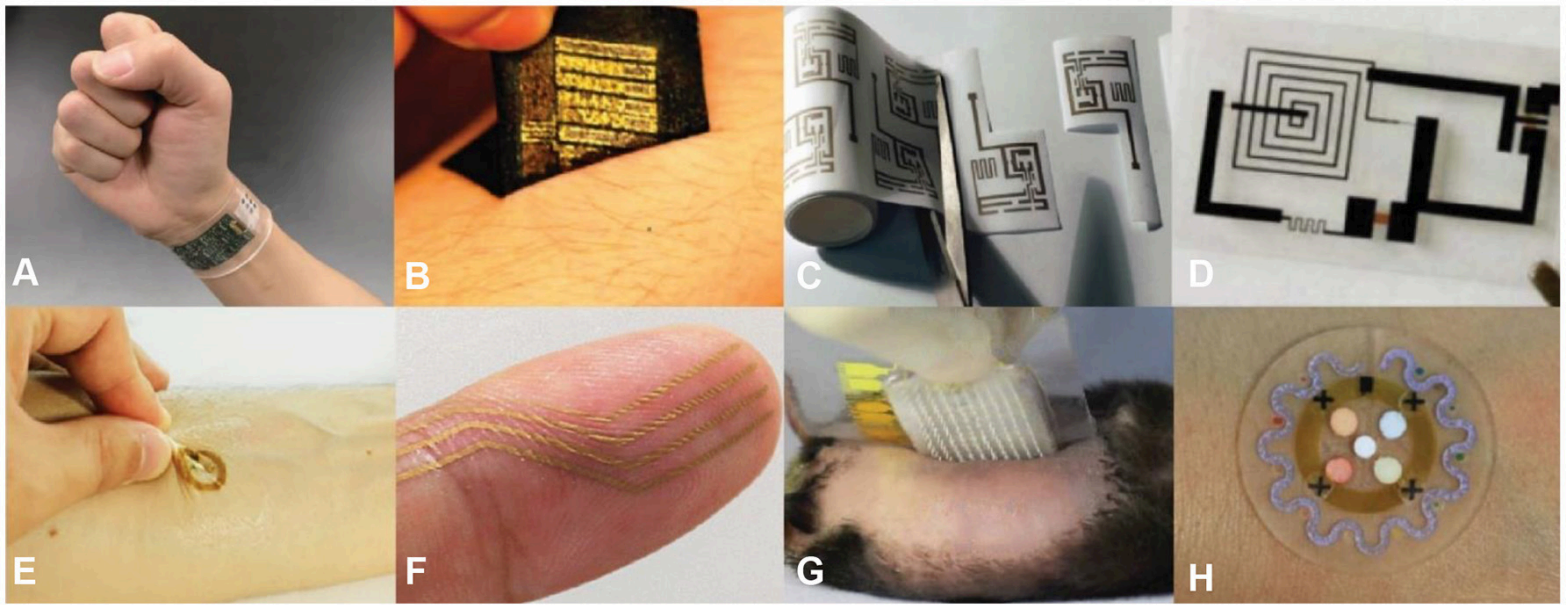
Various material substrates **(A–D)** and platforms **(E–H)** that have been developed for flexible biosensors. Material substrates include: **(A)** polymer, **(B)** textile, **(C)** paper, and **(D)** transient. Platforms include: **(E)** epidermal, **(F)** nanomesh, **(G)** microneedle, and **(H)** microfluidics. Reprinted with permission from: **(A)** Gao et al. (2016), **(B)** Jang et al. (2014), **(C)** Siegel et al. (2010), **(D)** Hwang et al. (2012), **(E)** Kim et al. (2016b), **(F)** Miyamoto et al. (2017), **(G)** Lee H. et al. (2017), **(H)** Koh et al. (2016).

Biosensor technology is advancing at an exponential rate. Engineering flexible biosensors capable of monitoring physiological information and assisting in proper treatment while laminating the device on the skin or an organ are technologically possible due to current advancements in the field (Kim et al., 2011; Webb et al., 2015). However, current wound care technology has been limited to laboratory testing, and no wearable POC type sensors are available for treatment alternatives. Flexible sensors have been fabricated through various microfabrication process to create a thin, stretchable, and flexible device that is mechanically compatible with the curvilinear surface of the biological system (O'Connor et al., 2016). Similarly to the wound dressings, the design of the biosensor for wound monitoring must be biocompatible, provide free fluid and gas flow, flexible, and stretchable. Therefore, the selection of substrate material, macro, and microstructure of substrate for an optimal wound sensor are crucial in the development to achieve an efficient device. Similar to the skin's elastic moduli (100–150 kPa) (Hattori et al., 2014), thin and flexible polymeric substrates exhibiting relatively low moduli (~60 kPa) (Zhang, 2016) accommodate any applied strain. Materials such as polymer, textile, paper (cellulose), and biodegradable (i.e., transient material) have sought to make for compelling substrate options (Figures 2A–D) for advanced wearable electronic devices. The system has been integrated on various platforms for intimate bio-integration including the form of epidermal, nanomesh, microneedle, and microfluidics (Figures 2E–H). Various materials and platforms for flexible biosensors offer solutions for creating a wound monitoring microsystem. Recent research that directly correlates to wound monitoring electronics is presented, however, we understand that the field is growing rapidly. Therefore, recent in-depth advancements in wearable sensor technology can be found elsewhere in review papers (Rogers et al., 2016; Gao et al., 2017; Heikenfeld et al., 2018).

### Materials for the substrate of the flexible biosensors

Polymer films, such as polyethylene terephthalate (PET) and polyimide (PI), are the staple materials for wearable technology because they form the skin to sensor contact primarily due to their high flexibility. For transparent flexible electronics, PET constitutes one of the commonly used substrates exhibiting robust mechanical properties (moduli 3.5–11 GPa), solvent resistant properties, tolerance to temperature (Tg ~100°C) (An et al., 2017), and biologically inert without cytotoxicity. Thus, PET substrates, often utilized for the application in optoelectronics, require transmission of electrical conductions and light through the substrate medium. Additionally, coating PET with indium tin oxide (ITO) develops PET into transparent electrodes, capable of optoelectronic applications (e.g., displays and photodiodes) (Zardetto et al., 2011). While the hydrophobic nature of PET creates difficulty in providing free gas and fluid flow through the substrate, chemical and physical modifications have been used to improve PET's hydrophilicity to some extent (Gotoh et al., 2011). Furthermore, the surface treatment of chemical modification has shown promising results in lowering the contact angle and increasing surface energy without altering its transparent nature while improving its biocompatibility (Wiria et al., 2016). As such, PET substrates have shown great flexibility in wearable technology in the application of providing structure to gold electrodes and flexible printed circuit boards (PCBs) (Figure 2A) (Gao et al., 2016).

The most used substrate, PI films offers more rigidity while still maintaining flexibility (moduli 3.7–20 GPa), and higher glass transition temperature (Tg ~300°C) (An et al., 2017) which is more desired in high temperature fabrication methods (Chang et al., 2008; Yoo et al., 2010). PI films have been widely used in substitution for bulky conventional PCBs, to keep the device flexible, lightweight, and comfortable (Farooqui and Shamim, 2016). In contrast, polydimethylsiloxane (PDMS) (moduli 4.8 MPa) (An et al., 2017) offers greater stretchability which provides a polymeric substrate matching mechanical properties to the biological interface, such as skin. Of note, various types of ultrathin, flexible, and stretchable bioelectronics that are highlighted in biomedical monitoring (e.g., temperature, ECG, and EEG) reported by pioneer research groups, Rogers and Someya group, used PDMS as a substrate (Jeong et al., 2014; Matsuhisa et al., 2015; Kim et al., 2016b; Liu et al., 2016). A wearable device for continuous wound oxygen monitoring, developed by Mostafalu et al. was constructed from PDMS to provide an oxygen-selective membrane (Mostafalu et al., 2015).

Textile materials offer a mechanically rugged, reusable, breathable, and lightweight alternative to thick polymer materials. Aliphatic polyamides (90% nylon) and polyurethane-polyurea (10% spandex) provide sensor systems with physically tough, yet flexible and breathable support while providing an increase in strain. Of note, nylon fabric substrates have shown to be rugged and breathable substrates for noninvasive *in situ* monitoring of biopotentials (i.e., EOG, ECG, and EMG) (Jang et al., 2014). Such materials present strategies for the integration of soft and stretchable materials in thin electronics on elastic fabric substrates for high adhesive, washable, breathable, reusable, and long-term skin-mounted electronics (Figure 2B) (Jang et al., 2014). The mechanical stability is governed by the fabric, yielding enhancement in reusability, whereas substrates lacking fabric lead to mechanical failures during the removal process (Jang et al., 2014). Fabric substrates' nonplanar nature creates difficulties during the fabrication process, screen printing specifically, which generally required uniformly flat surfaces. Such irregular substrates require an alternative fabrication procedure that can accommodate contours and irregularities of fabric materials. Although some of these challenges can be overcome through soft-lithography fabrication techniques (Windmiller and Wang, 2013), a strategy of system integration, and withstanding bending radii as small as 160 μm are still an unsolved problem (Kang, 2016).

Unlike other substrates, paper offers a unique set of advantages that are completely distinct from polymers or other conventional materials. Paper substrates are highly accessible, inexpensive, and readily obtainable. Paper's inherent properties including flexible, light, thin, porous, breathable, and easily tear-able advances flexible electronics by making them an inexpensive disposable option compared to the alternatives. However, paper-based electronics have several limitations: (1) repeated folding results in thin circuit wires tearing, leading to circuit failure, (2) during laser engraving the stencil and paper contact makes it difficult to fabricate advanced pattern features of which require a dimension less than 50 μm (Siegel et al., 2010). Whiteside's group shows a fully functional circuit and microfluidic system constructed on a paper substrate (Figure 2C) (Nie et al., 2010a,b; Siegel et al., 2010; Lan et al., 2013, 2014). Additionally, Choi's group presents a paper-based microbial fuel cell capable of sustainable power from microbial metabolism (Mukherjee et al., 2013; Fraiwan and Choi, 2014; Fraiwan et al., 2014; Lee and Choi, 2015).

Remarkable modern engineering such as transient electronics is a new class of technology which enables the device to be fully reabsorbed into the body (bioresorbable) by the body's own fluids. Common biodegradable polymers used as substrate materials are polylactic acid (PLA), polycaprolactone (PCL), poly(lactic-co-glycolic acid) (PLGA), and silk. These electronics can fully degrade while maintaining a sensor performance for a specific time, which can be altered through the synthesis of the substrate polymer ratio concentration and circuit nanomembrane doping levels (Figure 2D) (Hwang et al., 2012; Kang et al., 2016; Yu et al., 2016). The re-absorbability alleviates any negative impacts that long-term implantation may have on the biological system. In contrast, few electronics are biodegradable, rather many have been shown to produce toxic reagents through the degradation process, which limits the available circuit material.

### Platforms for system integration of flexible electronics

As state of the art, flexible electronics are emerging at an abundant rate, rapid advancements in sensing platforms have paved the way for a new class of wearable technology. Epidermal platforms, often referred to as temporary tattoo electronics, give rise to a new emergence of next generation technology for flexible and stretchable electronics. The overall engineering of the system allows for a much thinner, lighter, and “skin-like” flexible device providing a nonirritating and long-lived interface with the human epidermis (Kim et al., 2011, 2012; Windmiller and Wang, 2013; Wang et al., 2015). The platform allows for an increase in signal resolution due to the decreased impedance from direct, conformal contact with skin while exhibiting system thickness of <30 μm (Jeong et al., 2013) and modulus 150 kPa (Lu et al., 2016) which is similar to the epidermis (160 kPa) (Lu et al., 2016). Additionally, the ultra-thin feature is capable of conforming to skin and stretch along with skin deformations. Polymeric biocompatible elastomer materials, such as PDMS, are used to engineer this type of technology. Applications rely on noninvasive biomedical evaluations including biopotential (ECG, Liu et al., 2016, EMG, Jeong et al., 2014, and EEG, Norton et al., 2015), temperature (Chen et al., 2015), heart rate (Imani et al., 2016), blood pressure (Kim et al., 2016b), oxygen saturation (Yokota et al., 2016), blood flow (Webb et al., 2015), and electrochemical biomarker detections (Kim et al., 2016a). Roger's group has shown to engineer various on-skin electronics for electrical (Yeo et al., 2013; Jeong et al., 2014), optical (Gao et al., 2014; Kim et al., 2016b, 2017), and chemical (Koh et al., 2016; Lee Y. K. et al., 2017) analysis utilizing the epidermal platforms for various clinical applications (Figure 2E) (Kim et al., 2016b). Wang's group introduce various electrochemical biosensors on the temporary tattoo platform for real-time detection of metabolites (e.g., glucose and lactate), ions, pH, and other biomolecules (Windmiller et al., 2012; Bandodkar et al., 2013, 2015; Guinovart et al., 2013; Kim et al., 2015; Imani et al., 2016).

Electronics that are designed for the skin have limited gas permeability which causes inflammation on the skin due to the trapping of biofluids and air at the heterogeneous interface. A newly developed nanomesh platform developed by Someya's group (Figure 2F) (Miyamoto et al., 2017) shows the thinness of nanomesh (fiber diameter 300–500 nm) and how it conforms to the groves in the skin, resulting in a comfortable, lightweight, and stretchable elctrode biosensor that decreases impedance, while allowing mass transport of biofluids and gas. The ultrathin and nanoporous structure of this technology allows for long-term accurate monitoring of vital signals without any discomfort or inflammation reaction at the skin. In the study, electromyography (EMG) biosignals strength was maintained under repeated elongation of 40% while exhibiting signal-to-noise ratio comparable to conventional gel electrodes (Miyamoto et al., 2017). However, the expensive electrode material (i.e., gold), physical properties, and complexity of fabrication process limit mass production, robustness for long-term monitoring, and wide use in biomedical applications.

In applications where the high sensitivity of biopotential determination is required, the skin impedance negatively affects the signal by rendering it weak and unstable. Although nanomesh and epidermal platforms try to minimize skin impedance by improving the electrode to skin contact through the fabrication of a thin and low modulus electrode, a high-resolution signal may be desired in clinical practice. The stratum corneum of the skin is responsible for the increased impedance at the skin surface. Microneedle platforms percutaneously insert through the stratum corneum to decrease the skin impedance with a painless application process. The microneedle electrode acquires the signal from the dermis allowing highly sensitive evaluation, a longer wear time and biofluids sampling for further chemical analysis. However, the major drawback is that during implantation the microneedle can be subjected to bending (Chen K. et al., 2016). Recently, microneedle electrodes integrated with thin, flexible electronics are reported by Lee et al. demonstrating a painless therapeutic system for a noninvasive, closed-loop glucose monitoring system with high accuracy (Figure 2G) (Lee H. et al., 2017). Microneedle electrodes, similar to other platform types, offer flexibility and thinness maintained by advanced materials, process engineering, and system integrations.

Microfluidics allows for the manipulation of hydrodynamics of fluids at a microscale channel to study a specific parameter, engineered to replace macroscale assays and costly lab testing (Sackmann et al., 2014). A wearable microfluidic device for biomedical applications developed by Koh et al. (Figure 2H) (Koh et al., 2016) has demonstrated the harvesting of sweat, providing an overall analysis on key biomarkers (e.g., metabolite, pH, and chloride), sweat volume, rate to evaluate athletic performance, and monitor overall health status. The skin-mountable microfluidic device utilizes perspiration pressure to pump sweat into soft microfluidic networks where chemical analysis can be performed via colorimetric analysis. Additionally, designs of microfluidics with capillary bursting valves advances time-dependent sweat analysis monitoring of biomarkers and secretory fluidic local pressure (Choi et al., 2017a,b; Kim et al., 2018). Further, Choi et al. advanced microfluidic devices by improving sequential analysis (~8.5–18 min) while evaluating volumetric precision or local perspiration pressure (Choi et al., 2017a,b), in features of chrono-sampling of sweat, where newly generated sweat can be analyzed over time. Major limitations of the microfluidic system include limited temporal resolution and biofluids storage time due to vapor permeability of PDMS need to be overcome.

### Fabrication

Microfabrication techniques allow for the development of various platforms for advance flexible bioelectronics. Systems can be tailored to specific parameters based on the requirements of a device's design and biomedical application. While the number of different techniques is substantial, this review details those based on relevance, impact, overall performance, and potential emerging technologies in wound monitoring (Table 2).

**Table 2:** Common fabrication methods of flexible electronics.

**Category**	**Rate of production**	**Product resolution**	**Product materials**	**Advantages**	**Limitations**
**PRINTING**					
Ink-jet^a^	0.3–80 mm s^−1^	15–100 μm	Polymers, carbon nanotubes, colloids	Cheaper, maskless, reduced waste, scalable	Speed-consistency tradeoff, material restrictions
Screen^b^	0.01–1.6 mm s^−1^	30–100 μm	Polymers, carbon nanotubes, colloids, metals	Cheaper, scalable, roll-to-roll	Mask required, wasteful
Direct Laser^c^	10–100 mm s^−1^	100–450 nm	Polymers, hydrogels, proteins	Reproducible, 3D objects rendered in real time	Low productivity
Transfer^d^	N/A. No rate limiting step	10–200 nm	Metals, carbon, polymers, colloids, inorganic semiconductors	Versatile material choice, low resolution	Large variability in performance based on materials
**LITHOGRAPHY**					
Photo^e^	3–100 s per contact, varying other setup	0.5–5 nm, depends on source wavelength	Polymers, metals	Consistent, versatile settings	Long setup, mask required, cannot modify surface properties
Soft^f^	7–48 h curing time based on temperature	0.03–1 μm	Polymers, colloids, sol-gels, salts, metals	Cheaper, 3-D structure production	Difficult to reproduce, low productivity
Laser engraving^g^	200 mm s^−1^	0.05–1 mm	Polymers, plastics, metals, glass, wood	Maskless, wide material application	Overheating of materials, poor resolution
Soft-Hard Integration^h^	N/A. no rate limiting step	10–50 μm	Metals, polymers, inorganic semiconductors	Microfluidic suspension of hard electronics	Only for planar electronics

a *Singh et al. (2010) and Khan et al. (2015)*.

b *Khan et al. (2015)*.

c *Selimis et al. (2015)*.

d *Carlson et al. (2012) and Yoon et al. (2015)*.

e *Xia and Whitesides (1998); Bratton et al. (2006), and Khan et al. (2015)*.

f *Xia and Whitesides (1998) and Qin et al. (2010)*.

g *Gabriel et al. (2014)*.

h *Xu et al. (2014)*.

Photolithography has been applied for preparing a wide range of electronics. Briefly, photolithography yields micro-sized structures of active components for electronics and an array of modules using photoresists (UV responsive polymers) (Madou, 2002). The resolution in photolithography is defined by the wavelength of light exposure and the thickness of the photoresist layer. Flexible electronic materials such as PET and PI films have been applied in photolithography since the technique requires materials to exhibit dimensional stability through extreme temperatures (~10–573 K), pressures (in vacuum states), and various wet and dry etching procedures. For example, these films are placed on low-depth rigid substrates during processing, such as silicon, and adhere to a layer of poly (methyl methacrylate) (PMMA). After completion, the PMMA can be dissolved in acetone, separating the silicon from the final film design (Iberi et al., 2015). Photolithography has application in the development of various electronics, from complementary metal-oxide semiconductors (CMOSs) (Ooka et al., 2015), field effect transistors (FETs) (Yao et al., 2013), thin film transistors (TFTs) (Geier et al., 2015), temperature and pressure sensors (Marsi et al., 2015), LED (Chen M. et al., 2016), dielectric, semiconductors, and conductors.

The technique of soft lithography uses a combination of steps to design 3D patterns applied to flexible substrates, such as micro-channels and reservoirs. The master design is produced using conventional photolithography techniques, and PDMS—soft silicone-based elastomeric polymer—is coated over the master and cured (Whitesides et al., 2001). Following the cure of PDMS, the replica can be removed from the master design, and embedded within its structure as the same micro-designs that were developed by the master. Soft lithography is used in a wide variety of applications, from microfluidics (Koh et al., 2016; Choi et al., 2017a,b) and lab-on-a-chip technology (Dy et al., 2014) to flexible imprinted thermal characterization sensors (Gao et al., 2014).

Inkjet printing is the process of drop-casting micro-droplets of material while controlling its volume and evaporation rate (Calvert, 2001). An ink composed of desired material fills a chamber attached to a nozzle that is controlled via piezoelectric effects. With an applied current, the shockwave from the piezoelectric material induces the ejection of an ink droplet through the nozzle, with droplet diameter and thickness dependent on the current. When the droplet settles on the material of interest, the solvent evaporates in a controlled manner, leaving the solute product on the material (Singh et al., 2010). Inkjet printing does not require a mask for design, increasing fabrication versatility. However, issues arise from the ink's lack of adhesion to a substrate. Recent work has been done to overcome this limitation using molecular adhesion layers composed of 3-mercaptopropyltriethoxysilane (MPTES) or 3-aminopropyltriethoxysilane (APTES) (Jeong et al., 2010). To enable smaller resolution, recent methods have employed laser sintering, reducing thermal change and damage to the ink particles (Seung et al., 2007). Applications include fabricating conductive nanoparticles (Shen et al., 2014) and printed graphene structures (Secor et al., 2013).

In screen printing, ink is patterned across a screen mask onto a substrate using a squeegee. The ink pattern on the substrate will correspond to the mask pattern (Khan et al., 2015). Components are generated quickly yet accurately, with repeatability for roll-to-roll production. Soft materials and viscous inks have more success, while still generating designs with 100 μm resolution (Khan et al., 2015). Examples of screen printing in flexible electronics include soft components in UV dosimeters (Araki et al., 2017), poly(butylene adipate-co-terephthalate) (PBAT) designs (Abellán-Llobregat et al., 2017), and all components in screen-printed electrodes (Barton et al., 2016).

In transfer printing, PI film encapsulates electrical components on a silicon wafer and is transferred to a soft substrate through exchangeable adhesion forces. Conventional microfabrication techniques can then be used, such as photolithography (Lu, 2013). When completed, an adhesive layer (PDMS, water-soluble tape, or heat responsive tape) is placed over the structures and applied with pressure. With the proper velocity and motion, manual peeling of the adhesive membrane can also remove the intact electrical structures from the wafer (Rogers et al., 2010). The structures can then be placed facing an elastomeric material (e.g., PDMS), and the adhesive layer can be removed. The final product is the electronic devices fabricated through rigid techniques incorporated on a soft substrate that mimics criteria for flexible distinction (Rogers et al., 2010). Applications of this method have been used in designing electronics that conform to skin movement (Kim et al., 2011).

With direct laser writing (DLW), a polymer of interest is exposed to an ultra-fast laser focused through a transparent membrane. This focusing results in two or more photons absorbed simultaneously and can control the depth and location of where the polymerization occurs, rendering 3D objects without layer-by-layer fabrication or masks (Selimis et al., 2015). While a time-consuming process, it functions within the nanoscale resolution, a leading system for constructing flexible systems such as polyimide material generation in supercapacitors (In et al., 2015) and graphene-based electronics (El-Kady and Kaner, 2014).

Laser engraving is a form of non-contact fabrication employing a high-energy CO_2_ laser beam to cut through a material. The beam can either be continuous or have pulses fired in rapid succession (Gabriel et al., 2014). For most models, the stage is stationary while the laser beam moves to cut the design, or the stage moves underneath the laser beam. While limited in resolution due to beam radius, it remains versatile in material usage, with high compatibility and a stage with a larger surface area for faster production (Gabriel et al., 2014). Examples in flexible electronics include “cut-and-paste” manufacturing of epidermal sensors designed by Lu's groups (Yang et al., 2015; Wang et al., 2018).

Many current electronic components are limited to hard substrates and have been limited to transform into functional, flexible electronics. Xu et al. enabled the strategy to combine rigid circuits with an apparatus that mitigates the differences in mechanical forces on flexible electronics (Xu et al., 2014). The hard components of the circuit are connected through a flexible serpentine connecting wire. These structures are sealed in an elastomeric pouch filled with dielectric gel, serving the purpose of increased electrical conductivity and force dissipation. The pouch can then be attached to a moving substrate, and the hard components can withstand the stresses and strains corresponding to most flexible electronics (Xu et al., 2014). While significant in its ability to incorporate commercially available electronics, its size is limited by the commercial products' parameters which are large for most biological applications.

## Chemical and physical analysis using the flexible biosensors

To provide accurate and precise wound monitoring, the flexible biosensors can be coupled with detecting notable biomarkers. The biomarkers critical to wound status would provide clinical and continuous information if it could be analyzed with a point-of-care system. Glucose, lactate, pH, oxygen, interleukin-6, as well as temperature and blood pressure values at the wound site can determine wound healing progression and stages of inflammation. Current research focus also includes developing sensors with bacterial selectivity detecting bacterial colinization. Significant biomarker physiology is emphasized and discussed in the Wound Repair Physiology section above. This section summarizes recent work in more accurate, viable, and flexible biosensing technology in biofluids, potentially for chronic wound monitoring (Table 3).

**Table 3:** Common biomarkers evaluated by biosensors for wound monitoring.

**Type of marker**	**Indicator of:**	**Normal values**	**Illness values**	**Main mechanism of measurement**
**CHEMICAL**				
Glucose^a^	Insulin deficiency, diabetes mellitus	3.9–7.8 mM in blood	>7.8 mM in blood	Chronoamperometry, absorptiometry
IL-6^b^	Inflammation, elevated metalloproteinases	0 pg/μg on skin 0–2.4 pg/mL in blood	1.27 ± 1.7 pg/μg at wound >2.4 pg/mL in blood	ELISA, western blot
Lactate^c^	Hepatic disease, tissue hypoxia, hemorrhage, sepsis	0.5–1.5 mM in blood 1.0–3 mM on skin	>1.5 mM in blood >7 mM at wound	Chronoamperometry
pH^d^	Infection, acidosis, enzyme degradation rate, collagen deposition, fibroblast activity	4–7 pH of skin	7.15–8.90 pH at wound	Chronoamperometry, optical transitions
Oxygen^e^	Metabolic activity, apoptosis, carbon monoxide	30–50 mmHg pO_2_ on skin 50–130 μL in blood 97% hemoglobin binding	5–20 mmHg pO_2_ in exudate 50–130 μL in blood <97% hemoglobin binding	Fluorescent sensing, clark-type electrochemical cell
**PHYSICAL**				
Temperature^f^	Inflammation, metabolic activity	31.1–35.4°C of skin	1.11°C increase at wound	Thermometer, thermogram, thermistor
Blood Pressure^g^	Hypertension	<115 mmHg systolic <80 mmHg diastolic	>115 mmHg systolic >80 mmHg diastolic	Strain gauge, auditory

a *Chen et al. (2013)*.

b *Beidler et al. (2009); Huang et al. (2013); Tsuneyasu et al. (2014)*.

c *Löffler et al. (2011) and Rathee et al. (2016)*.

d *Lambers et al. (2006); Gethin (2007); Morris et al. (2009), and Santos et al. (2014)*.

e *Schreml et al. (2010); Baura (2012b), and Luo et al. (2017)*.

f *Karlsbad and Kopp (1991); Ng (2009); Fierheller and Sibbald (2010), and Baura (2012c)*.

g *Lawes et al. (2008) and Baura (2012a)*.

### Chemical biomarkers

#### Glucose

Through glycolysis, glucose is broken down to yield ATP, driving numerous thermodynamically unfavorable reactions across the body. Normal concentrations range from 3.9 to 7.8 mM in the blood (Chen et al., 2013), where higher levels indicate insulin deficiency. With abnormal insulin levels, diseases such as diabetes mellitus can occur, resulting in increased glucose sensitivity. For patients who have diabetes, the moment a skin injury occurs, specifically in the lower extremities, there is a high risk for amputation (Brem and Tomic-Canic, 2007). Wound healing deficiencies resulting from high glucose levels cause impairments in growth factor production; angiogenesis, macrophage function, collagen deposition, scar strength, keratinocyte, and fibroblast aggregation at the wound (Brem and Tomic-Canic, 2007). Clinically, diabetic patient's glucose levels are closely monitored and regulated accordingly to mitigate the negative symptoms of hyperglycemia. Therefore, glucose is one of the most common biomarkers studied since diabetes mellitus affects millions of people and is accommodated with detrimental symptoms.

Most of the glucose measuring techniques rely on electrochemical chronoamperometry. Levels of a targeted analyte on the working electrode generate a direct current over time, functioning for enzymatic and nonenzymatic glucose sensing. Enzymatic glucose sensing requires immobilized glucose-oxidase (GOx) on the electrode surface breaking down glucose into gluconic acid (Wang, 2008). Various immobilizing assays are composed of conductive nanomaterials, binding to the enzyme while also improving electronic transfer. Current examples include nanometal oxides (Rahman et al., 2010), graphene-carbon nanotube composites (Yu et al., 2014) and polyaniline-modified platinum nanoparticles for blood glucose sensing (Wu and Yin, 2011). Sweat glucose sensing has been researched by Lee et al. using flexible epidermal sensors on PDMS corrected with temperature, pH, and humidity measurements (Lee H. et al., 2017). For another example, the screen-printed epidermal tattoo sensors encapsulated in agarose hydrogel measured interstitial fluid glucose levels through reverse iontophoresis on the skin's surface (Bandodkar et al., 2015). Enzymatic electrochemical limitations include a lack of long-term stability, as GOx denatures over time. Non-enzymatic sensors address this issue, by measuring the direct oxidation of glucose to gluconic acid on a novel metal working electrode (Pt or Au) including Cu_2_O encapsulated in graphene nanosheets (Liu et al., 2013), 3D porous Ni foam nanoparticles (Lu et al., 2013), and Ni-Al layered double hydroxide/single-walled carbon nanotubes (SWCNT)/graphene nanocomposite layered on Au nanoparticles (Fu et al., 2015). While sustainable across longer periods of time, the process is irreversible, the absorption of oxidation intermediates lowers the activity of the electrode, selectivity of detecting glucose is lower as other interferences can be oxidized in the same range as glucose, and often requires basic condition (pH >10) for greater sensor performance (Chen et al., 2013). Both systems sustain an approximate 0.25 mM limit of detection (LOD) (Chen et al., 2013).

Optical measurements for glucose have been developed over recent years, and are less invasive, able to reach less accessible regions in the body and use different component mechanisms (Steiner et al., 2011). The exploited mechanism in optical measurements is having a chemical undergo photon-absorption intensity changes when bound with glucose, through fluorescence, infrared spectroscopy, optical coherence tomography, and more (Steiner et al., 2011). Current examples include GOx encapsulated with sol-gel doped in crystalline iridium(III)-containing coordination polymers (Ho et al., 2014), fluorescent hydrogel fibers (Heo et al., 2011) and injectable hydrogel microbeads (Shibata et al., 2010) for continuous *in vivo* monitoring, and silica nanoparticles encapsulating organic dye on PMMA for glucose monitoring in tear-based contact lens sensors (Zhang and Hodge, 2013). Qu et al. also reported using 3-aminobenzeneboronic acid functionalized graphene quantum dots as binding agents for fluorescence glucose monitoring (Qu et al., 2013). Limitations with optical glucose sensing are a lack of specificity for glucose, and many have yet to be successful with *in vivo* testing (Steiner et al., 2011). Optical glucose sensing LOD sets at approximately 80 μM (Steiner et al., 2011).

#### Lactate

During the anaerobic activity, the pathway through which we receive energy is modified due to oxygen deficiency, producing lactate (Rathee et al., 2016). Standard levels range from 0.5 to 1.5 mM in blood, but persistent concentrations above 1.5 mM represent hyperlactatemia (Rathee et al., 2016). Generally, normal tissue lactate levels are around 1–3 mM, and fibroblasts become impaired with lactate concentrations above 7 mM at the wound site (Löffler et al., 2011). Additionally, outside of normal blood lactate concentrations, this can indicate more serious conditions further impeding the wound healing process (e.g., lactic acidosis, lactic sepsis, or tissue hypoxia) (Rathee et al., 2016).

Compared to glucose, lactate can be monitored chronoamperometrically. Gao et al. demonstrate lactate oxidase encapsulated by chitosan-SWCNT matrix on the flexible gold electrode for sweat measurements (Gao et al., 2016). Other recent developments include screen printed lactate oxidase-chitosan mixture combined with polyvinyl chloride-tetrahydrofuran solution on the flexible polyester sheets (Imani et al., 2016), lactate oxidase immobilized in a sol-gel matrix encapsulating reduced graphene oxide-gold nanoparticles (Azzouzi et al., 2015), and screen printed carbon Ag/AgCl inks modified with carbon nanotube-tetrathiafulvalene lactate oxidase suspension for use in temporary tattoo sensor (Jia et al., 2013). Lactate electrochemical LOD reaches approximately 0.05 mM (Gao et al., 2016). Lactate sensors experience complications from oxygen fouling and enzymatic instability over time, similar to glucose electrochemical sensing. Other methods have been developed for detection of lactate. Hu et al. reports using cupric oxide nanoparticles to activate terephthalic acid via hydrogen peroxide as an optical fluorescent lactate sensor (Hu et al., 2014). Kazakova et al. modified calcium carbonate microparticles with lactate oxidase and absorbed fluorescent dye-labeled poly(allylamine) hydrochloride and activated poly(sodium-4-styrene sulfonate) to construct polyelectrolyte optical sensors (Kazakova et al., 2013). Optical lactate sensing corresponds to a 0.34 μM LOD (Hu et al., 2014). A colorimetric analysis presented by Koh et al. utilized lactate and co-factor NAD^+^ (nicotinamide adenine dinucleotide) enzymatic reactions by lactate dehydrogenase and diaphorase producing a color change of a chromogenic reagent (Koh et al., 2016). The microfluidic device was capable of detecting lactate concentrations ranging from 1.5 to 100 mM, an applicable range expected from sweat.

#### pH

Hydrogen ion concentration is a critical biomarker in determining a healthy or diseased state. Chronic wounds exhibit a pH around 7.15–8.90 at the wound bed, however through tissue repair it is more favorable to have a more acidic environment (4–7 pH) to decrease abnormal collagen formation, increase fibroblast activity, slow metalloproteinase enzyme degradation rates, and yield a harsh environmental condition to decrease bacterial viability and infection at the site (Lambers et al., 2006; Gethin, 2007). Consequentially, a more basic pH environment signals abnormal healing conditions and can indicate alkalosis, or pathogenic infection (Gethin, 2007).

Chronic wound pH is commonly monitored through a potentiometric method which is simple and provides a portable electrochemical technique (Santos et al., 2014). Applications include using four-channeled gold and silver electrodes on a microfabricated flexible PET film (Yun et al., 2017). Gold has also been applied with tungsten oxide (WO_3_) on the polyimide film, constructed to measure or influence pH (Santos et al., 2014). Bandodkar reports using screen-printed ion-selective carbon electrodes (ISEs) on the temporary transfer tattoo paper for epidermal pH detection (Bandodkar et al., 2013). However, limitations arise as the leaching ionophore from ISEs is cytotoxic.

Alternative methods in detecting pH at the wound site exploit changes in the optical appearance of materials in response to specific pH levels. Many indicators and dyes change color under different pH conditions, where the most common for physiological purposes being bromocresol purple (BCP), phenol red, and naphtholphthalein. This colorimetric approach immobilizes dye on a flexible substrate, using various recording techniques (Morris et al., 2009). Morris et al. used BCP to measure pH levels in sweat on a silicone-infused textile device while utilizing the optical electronic systems (Morris et al., 2009). Optical pH sensors exhibit a resolution of 0.01 pH (Wencel et al., 2013). Curto et al. presented a microfluidic device constructed of PMMA, where fresh sweat is continuously harvested, and detection is indicated by chromic pH-sensitive dyes (Curto et al., 2012). The microfluidic device was reported to provide an operational lifetime of 135 min. Limitations arise as the sensor is only capable of detecting pH levels once. However, such shortcomings can be overshadowed by the device being easily fabricated and modified accordingly to provide more extended real-time measurements (Curto et al., 2012). Nakata et al. demonstrated the first proof-of-concept operation of a real-time, simultaneous sweat monitoring, pH and temperature through the ion-sensitive field-effect transistor (ISFET) on a flexible PET film (Nakata et al., 2017).

#### Oxygen

Attention to oxygen sensors is focused on arterial oxygen concentration, which is compositely determined by hemoglobin concentration and partial pressure of dissolved oxygen in the blood (Vaupel et al., 1989). Under normal conditions, hemoglobin accounts for 97% of oxygen transport through the bloodstream, while 3% is dissolved in blood (Baura, 2012b), corresponding to a range from 50 to 130 μM (Pita et al., 2013). The partial pressure of dissolved oxygen in systemic arterial blood ranges typically between 95 and 100 mmHg, while the systemic venous blood ranges from 0 to 10 mmHg (Baura, 2012b). During persistent inflammation, the partial pressure of dissolved oxygen (pO_2_) in wound exudate ranges from 5 to 20 mmHg, whereas healthy tissue exhibits levels from 30 to 50 mmHg (Schreml et al., 2010). Abnormal oxygen levels can indicate large levels of cell apoptosis, carbon monoxide poisoning, changes in pH, and promote overexpression of proinflammatory cytokines (e.g., IL-6, IL-1β, and TNF-α) (Schreml et al., 2010; Baura, 2012b).

Contrasting to other analytes, optical oxygen sensing is mainstream. Unique oxygen optical characteristics are exploited, mainly their absorption differences between hemoglobin bound to oxygen (940 nm) vs. unbound (660 nm) (Baura, 2012b). Pulse oximeters have been developed to record these differences, indicating blood oxygen saturation. Light deriving from LED's are pressed against the finger, with the returning signal recorded by an optoelectronic sensor. While a noninvasive system, it is prone to optical interference from hemoglobin in other configurations (bound with carbon monoxide) or enzymes such as bilirubin (Baura, 2012b). Current works in pulse oximetry facilitate LED's and optoelectronic sensors to construct on the thin and flexible substrates capable of conformal contact with the skin (Lochner et al., 2014; Kim et al., 2016b). Other optical oxygen measuring includes the systems utilizing luminescent Pt(II) porphyrin silicate complex (Wang et al., 2014), PDMS nanofibers with tris(4,7-diphenyl-1,10-phenanthroline) ruthenium(II) encased in polycaprolactone (Xue et al., 2014), and fluorinated xerogel with palladium (II) meso-tetrakis(pentafluorophenyl)porphyrin quantum dots (Chu and Chuang, 2015). Optical oxygen sensing LOD rests at 0.1 μM of hemoglobin (Baura, 2012b).

Recent oxygen sensors have been developed using chronoamperometry. Luo et al. reports encasing a gold electrode in a PDMS-solid state Nafion electrolyte composite for oxygen dissolved in a microfluidic system (Luo et al., 2017), while Pita et al. include bilirubin oxidase immobilized on gold nanoparticles modified with diazonium-thiol mixed monolayers (Pita et al., 2013). Electrochemical oxygen sensing maintains a 0.2 μM LOD (Luo et al., 2017).

#### Interleukin-6

IL-6 is a cytokine that is correlated to markers of the proinflammatory response (Gabay, 2006). Much of the information on IL-6 and its role in the body can be found in the Wound Healing section. Elevated IL-6 levels have been shown to degrade ECM, diminish cell migration, and maintain the innate inflammatory wound healing response (Eming et al., 2014). IL-6 levels in the blood range from zero to 2.4 pg/mL, whereas in tissue, IL-6 is absent (Beidler et al., 2009; Huang et al., 2013). Abnormal levels in the blood are seen greater than 2.4 pg/mL, and at the wound 1.27 ± 1.7 pg/μg which serve as indicators of inflammation and elevated metalloproteinases (Bastard et al., 2000; Beidler et al., 2009; Huang et al., 2013). Many standards for immunosensing use techniques such as enzyme-linked immunosorbent assay (ELISA) or Western Blot, but both have limitations scaling to a portable, flexible substrate for point-of-care diagnostics.

Amperometric techniques have shown successful analyzing IL-6. A dual amplification method reported with a gold nanoparticle-poly-dopamine and synthesized horseradish peroxidase-antibody functionalized carbon nanotubes (Wang et al., 2010). Fan et al. also reported using an ITO electrode covered with TiO_2_/CdS/CdSe dual co-sensitized structure (two or more sensitizers accompanied with a photoactive material), using glutaraldehyde and chitosan to immobilize the anti-IL-6-antibody in a photoelectrochemical sensor (Fan et al., 2014). Malhotra et al. used SWCNT forests combined with multilabel detection capable of measuring a broad range of IL-6 concentration. Densely packed SWCNTs attached with capture antibodies (Ab_2_) bind to the antigen and then enzyme label secondary antibody (Ab_2_) bioconjugate interacts to the antigen (Malhotra et al., 2010). For wound healing applications, results in dynamic range and prolonged activity still perform worse than clinical immunoassays, although IL-6 LOD reaches values as low as 1.0 pg/mL, within biological testing restraints (Wang et al., 2010).

IL-6 has also been measured using various other systems. Mok et al. articulated using microfluidic setups, employing anti–IL-6–conjugated microbeads and recording the number attached to a gold electrode for impedance sensing (Mok et al., 2014). Mandel et al. used soft lithography designed PDMS microfluidic channel functionalized with biotinylated monoclonal antibodies, coupled to a one-dimensional photonic crystal resonator array with planar photonic crystals to yield an optical IL-6 sensor (Mandal et al., 2009). Future optimizations are necessary before biological application, as current models only reach a 50 pM LOD (Mok et al., 2014).

### Physical biomarkers

#### Temperature

Temperature is one of the most commonly recorded physiological markers in the body. Rates of many enzymatic reactions are temperature dependent, emphasizing the importance of normalized tissue temperature. The range for the temperature at the skin is from 31.1 to 36.5°C (Ng, 2009). At the wound site a prolonged temperature increase of at least 1.11°C could be due to infections and metabolic activity changes, (Karlsbad and Kopp, 1991; Fierheller and Sibbald, 2010; Baura, 2012c). Numerous methods are used for measuring temperature, but this review will focus on those which can apply for flexible, noninvasive substrates.

Many common sensors extort the principle of metals changing resistance in response to temperature. Webb et al. used this property and applied it to two systems: one was gold encased in polyimide serpentine microstructures fabricated using photolithography, and the other was multiplexed arrays of sensors based on PIN diodes formed by patterned doping of silicon nanomembranes, both of which were developed on the thin polymeric films for skin adherence (Webb et al., 2013; Hattori et al., 2014; Krishnan et al., 2017). Copper, used by Hattori et al. as micro-metal resistors, was engineered into an interconnected arrangement of ultrathin filamentary serpentine structure, laminated on a polyimide substrate (Hattori et al., 2014). The device was proven to adhere to the perilesional wound and provide accurate temperature and thermal monitoring (Hattori et al., 2014). In sophisticated thermal sensor/actuator arrays designs, impedance sensors can be incorporated into the similar device configuration and provide epidermal hydration sensing (Webb et al., 2016). Other materials, such as graphene (Trung et al., 2016), silver (Yamamoto et al., 2017), and nickel (Jeon et al., 2013) have been used in temperature sensing modalities.

The body irradiates heat in the form of infrared (IR) electromagnetic waves. Measure IR power using pyroelectric materials generate a voltage due to a change in temperature correlates with body temperature (Baura, 2012c). Ota et al. developed a customizable 3-D printed thermophile IR sensor, which utilized liquid metal microchannels of Galinstan, rendering it compatible with 3D printing. The base substrate, polyurethane, accounted for the flexibility of the device and lamination in the ear, reading IR measurements from the tympanic membrane (Ota et al., 2017). A dielectric pyroelectric material in an organic thin-film transistor, reported by Tien et al., was used a Ni-gated electrode encased in polyimide with dielectric poly(vinylidene fluoride) and trifluoroethylene (Tien et al., 2009). The device produced a linear response in an acceptable temperature range, proving its potential implementation into pyroelectric thermal sensors (Tien et al., 2009). Overall, noise is the most significant issue in IR temperature sensing, as it reaches a LOD of 0.005 K (Ota et al., 2017).

#### Blood pressure

Normal blood pressure levels are defined as having a systolic pressure (SP) below 120 mmHg and diastolic pressure (DP) lower than 80 mmHg (Chobanian et al., 2003). A chronic result of blood SP higher than 140 mmHg is hypertension, which is an illness that can negatively affect wound healing rate (Baura, 2012a). Hypertension and diabetes are interrelated diseases (Sowers and Epstein, 1995). Additionally, individuals afflicted with diabetes are twice as likely to develop hypertension in comparison to those without (Sowers and Epstein, 1995).

The flexible devices employ using a strain gage for measuring blood pressure. With a change in length of the underlying surface, a metric of the metal changes depending on its properties, which can be measured (Baura, 2012a). Gong et al. developed gold nanowires encased with a tissue paper in PDMS sheets integrated with interdigitated electrode arrays to measure changes in electrical resistance as a function of changes in length (Gong et al., 2014). In addition, Cohen et al. used photolithography to generate parallel carbon nanotube percolation electrodes separated by a dielectric silicone elastomer for capacitive strain measurements (Cohen et al., 2012). Another report includes nanofiber assembly of regioregular poly(3-hexylthiophene) were electrospun between gold electrodes, where a change in contact between the fibers generates a change in system resistance (Gao et al., 2012). Yao et al. report using fractured microstructure design in a graphene-nanosheet-wrapped polyurethane (PU) sponge to piezo-electrically measure resistance change from the strain (Yao et al., 2013). The zirconate titanate utilized as the piezoelectric sensor for subcutaneous blood pressure monitoring showing fast response times (~0.1 ms) and high sensitivity (~0.005 Pa) (Dagdeviren et al., 2014). While accurate within blood pressure scale and inexpensive from the micro-level material used, strain gage sensors must be placed specifically on the body to function, and are fragile in long-term use (Gong et al., 2014). The reported sensors show a LOD of around 0.001 strain change (Gong et al., 2014).

### Bacteria biosensor

In wound health prognosis, bacterial identification is important to assess the status and health of the wound. A chronic wound that becomes overwhelmed with bacterial colonization deprives the wound of oxygen and nutrients, and prolong the inflammatory phase, thus further extending the wound healing time (Zhao et al., 2013). Early detection of bacterial colonization allows for clinicians to provide a proper treatment regimen for specific antibiotic therapies. Conventional identification of bacteria utilizes culture-based methods, which is time-consuming (Chen Y. et al., 2016). Current research efforts, thus, have been focused on developing rapid screening methods by portable biosensors. Biosensors developed for bacterial identification rely on nucleic acid oligonucleotides with an array platform, and enzymes (Lim et al., 2015). Although current microorganism biosensors can detect a wide range of organisms, they still suffered from lack of sensitivity and selectivity (Table 4) (Lim et al., 2015). Additionally, the Point-of-Care (POC) devices providing immediate onsite results are in demand for bacterial identification in a clinical setting and developing countries to eradicate the need for labor-intensive laboratory evaluation.

**Table 4:** Various biosensors designed for microbial detection.

**Microbial sensor**	**Biomarker measuring mechanism**	**Analytical measurement**	**Limit of detection**
*Pseudomonas aeruginosa*	Pyocyanin metabolite from wound exudate^a^	Electrochemical	56 CFU/mL
	*ecfX, ExoS, and ExoU* genes^b^	mLAMP, LFNAB, colorimetric	20 CFU/mL
	Phage binding to host^c^	Electrochemiluminescent	56 CFU/mL
*Staphylococcus aureus*	anti-*S. aureus* aptamer^d^	Potentiometric transducer	Covalent 8 × 10^2^ CFU/mL Non-covalent 10^7^ CFU/mL
	Proteolytic activity^e^	Colorimetric	7 CFU/mL

a *Sismaet et al. (2016)*.

b *Chen Y. et al. (2016)*.

c *Yue et al. (2017)*.

d *Zelada-Guillén et al. (2012)*.

e *Suaifan et al. (2017)*.

The two most common types of bacteria present in chronic wounds are *P. aeruginosa* and *S. aureus* (Hill et al., 2010). Specifically, *P. aeruginosa* is a common pathogen in nosocomial infections, allowing for high risk of chronic wound patients (Sismaet et al., 2016). Currently, the gold standard for *P. aeruginosa* detection is a microbiological technique based on plate counting of cultured bacteria (Yue et al., 2017). To overcome these obstacles, Sismaet et al. reported an inexpensive, disposable, electrochemical sensor capable of detecting pyocyanin, a redox-active quorum sensing molecule specific to *P. aeruginosa* in wound exudate (Sismaet et al., 2016). The biosensors screen-printed 3-electrodes setup on the mesh dressing analyzed pyocyanin with a detection limit of 56 CFU/mL and sensitivity of 71% and specificity of 57% in comparison to 16S rRNA sequencing which is a diagnostic standard. A device capable of visual detection through loop-mediated isothermal amplification (mLAMP) and lateral flow nucleic acid biosensor (LFNAB) has also developed to detect *P. aeruginosa*, reported by Chen Y. et al. (2016). The device utilized a capillary action through the overlapping paper membrane system and established a colorimetric signal with gold nanoparticles while specifically binding with bacteria. The sensor can detect low levels of *P. aeruginosa*, 20 CFU/mL with lifetime >1 month. Recently, Yue et al. introduce a novel approach for label-free detection using electrochemiluminescent (ECL) biosensor (Yue et al., 2017). The virulent phage (PaP1) specifically binding to *P. aeruginosa* was modified on the carboxyl graphene-coated glass carbon electrode to govern biological recognition, and ECL signal decreased upon interference electron transfer due to the formation of non-conductive microorganism. The sensor yielded high specificity and a limit of detection of 56 CFU/mL.

Unlike *P. aeruginosa*, the thick layer of polysaccharide produced by *S. aureus* provides challenges to engineer a reliable biosensor. Thus, there are limited biosensors that have been developed for analysis of *S. aureus* with unsatisfactory detection limits (Zelada-Guillén et al., 2012). Nonetheless, an anti-*S. aureus* aptamer biosensor to detect *S. aureus* has been developed by Zelada-Guillén et al. (2012). A network of single-walled carbon nanotubes (SWCNTs) was utilized as the potentiometric transducer with the anti-*S. aureus* aptamer. Through covalent functionalization, the limit of detection was 8 × 10^2^ CFU/mL which was accompanied by low sensitivity (0.36 mV/Decade), whereas the non-covalent method yielded a higher limit of detection (10^7^ CFU/mL) and higher sensitivity (1.52 mV/Decade). In another study, Suaifan et al. reported a colorimetric paper-based biosensor reacting to the affinity of magnetic nanobeads to the proteolytic activity of *S. aureus*, (Suaifan et al., 2017). The color of the sensor changed from black to gold when contacted with *S. aureus*. The device was capable of sensing limits as low as 7 CFU/mL. Although these sensors provide effective alternatives to the current technique, research of the efficacy of these new sensing methods and system integration on the wearable platform are still in demand to utilize the sensing strategies for wearable biosensors.

## Conclusion

Chronic wounds are a significant health burden for individuals and have a substantial financial impact on the health care system. New and innovative therapies are being devolved to improve upon the current standard of care. Clinically, wound assessment and diagnosis are based on laboratory testing, which is time consuming, labor intensive, costly, and does not consider the complex, changing wound environment. Additionally, modern treatment methods lack the versatility of tackling multiple wound symptoms and require frequent removal and reapplication which can disrupt the wound bed, cause painful removal, and increase the overall medical costs. Forthwith, a multifunctional device capable of improving the healing process, preventing infection, and continuously observing vital biomarkers at the wound site while providing a therapeutic closed loop system has the potential to provide patients with an improved treatment alternative. Important determination from the device includes pH, temperature, glucose, lactate, oxygen, infection feedback, and if a dressing change is needed. Ideally, the sensor would also be wireless and self-powered. Successful wound monitoring devices could decrease doctor appointments and costly lab testing associated with diagnosis and treatment of chronic wounds. Although, significant advancements have been achieved in the field, wound care treatment with a POC device has been limited to research and is not available clinically. Therefore, a device capable of wound surveillance and healing stimulation is highly desirable.

Improvements in flexible and stretchable biosensors will pave the way for advanced wound care technology integrated into tissue engineered substrates, capable of promoting increased tissue regeneration while simultaneously monitoring wound parameters. Transient electronics and a tissue engineered substrate would mitigate the immune response and promote enhanced wound healing, attracting vital cells through endogenously incorporated chemical signals (i.e., growth factors, differentiation factors, and cell adhesion molecules) (Singh et al., 2008) and providing a material matrix similar to the ECM.

In parallel with developing an *in situ* wound monitoring device, a synergistic research effort of incorporating therapeutic agents to assist in wound repair would improve the overall efficacy of the device. Pharmaceutical enhancements, distinct drugs able to not only increase and induce improved wound healing but also administer antibiotics to eradicate biofilms present on the wound bed. However, in instances where there is an absence of a bacterial biofilm the device should not elicit an antibiotic treatment since antibiotics have been shown to disrupt the natural healing process and promote necrosis of the tissue in wounds not cultured with bacteria (Lo et al., 2009). Therefore, accurate and precise wound monitoring is a crucial feature of a wound care device.

To fabricate such a device, future research should focus on the integration of sensors able to monitor physiological information and assist in providing proper treatment while worn. Individual works cited throughout this review have provided new solutions to engineering flexible and stretchable sensors to be used in the fabrication of a POC device applicable to improving wound care technology.

## Author contributions

MB and AK conceived and developed the framework for the outline and focus of the review paper. MB and BA drafted the article and created tables and figures. MB constructed the manuscript. MB, BA, and AK participated in the revision process of the article and gave final approval of the submitted version.

### Conflict of interest statement

The authors declare that the research was conducted in the absence of any commercial or financial relationships that could be construed as a potential conflict of interest.
